# Mitigation of Salinity Stress in Maize Seedlings by the Application of Vermicompost and Sorghum Water Extracts

**DOI:** 10.3390/plants11192548

**Published:** 2022-09-28

**Authors:** Khalid H. Alamer, Shaista Perveen, Abdul Khaliq, Muhammad Zia Ul Haq, Muhammad Usman Ibrahim, Bader Ijaz

**Affiliations:** 1 Biological Sciences Department, Faculty of Science and Arts, King Abdulaziz University, Rabigh 21911, Saudi Arabia; 2 Department of Agronomy, University of Agriculture, Faisalabad 38040, Pakistan

**Keywords:** salinity, sorghum water extract, organic amendment, antioxidants, chlorophyll

## Abstract

Abiotic stresses are important constraints limiting crop productivity worldwide. Salinity is one of the most devastating environmental factors restraining the production of crops. It is urgently needed to search for environmentally safe and sustainable approaches to mitigate the harmful effects of salinity on plants. Hence, applying vermicompost and low-dose aqueous extract of sorghum delivers a pragmatic solution to ameliorate the detrimental outcomes of salinity on maize seedlings (*Zea mays* L.). The experiment consisted of three factors, each at different levels, i.e., salinity (control, 6, and 12 dS m^−1^), vermicompost (control, 5, and 10%), and sorghum water extract (control, 1, and 2%). Higher salt stress negatively influenced the morpho-physiological traits of maize. Nonetheless, applying vermicompost and sorghum water extract at 10% and 2%, respectively, increased tolerance against salinity. The application of 2% sorghum water extract and 10% vermicompost significantly improved morphological characteristics, chlorophyll contents, activities of antioxidant enzymes, leaf and root K^+^/Na^+^ ratio, and K^+^ contents. It decreased Na+ concentration, H_2_O_2_, and malondialdehyde contents at higher salinity levels. It can be concluded that soil-applied vermicompost and foliar-applied sorghum water extract mitigates the adverse impacts of salinity by activating the antioxidant defense system, improving chlorophyll contents, and reducing the accumulation of Na^+^ under salinity.

## 1. Introduction

Soil salinization impacts over 800 million hectares of agricultural land worldwide [1]. The salt-affected area is continuously swelling owing to climate change events and irrigation with poor quality water [2]. Excessive salt in the soil system is deleterious for soil structure and plants. Ionic imbalance and osmotic stress are the outcomes of soil salinity; resultantly, plants face physiological drought and a deficiency of nutrients [3]. Furthermore, the accumulation of reactive oxygen species (ROS) increases in the plant, provoking nucleic acid injury, protein oxidation, and lipid peroxidation [2]. All the above-mentioned series of events in the plant system result in plant growth inhibition and yield loss. Crop yield losses caused by salinity are about 10% annually, with a monetary value of USD 27.3 billion [3].

Maize (*Zea mays* L.) is a member of the Poaceae family; it possesses premier nutritive value and ranks as the third most important cereal in Pakistan. Moreover, it plays a significant role in the country’s food demand and value addition to the economy. Maize plants are sensitive to soil salinity. Salinity has damaging effects through physiological, biochemical, and morphological mechanisms, resulting in poor stand establishment and stunted plant growth [4]. Poor plant performance under a saline environment results from plant hormone and nutrient imbalance, ion toxicity, osmotic stress, electrolyte leakage, disruption in cellular functions, and membrane damage [3,5]. Significant efforts are being put into mitigating the detrimental effects of salinity on crop plants. These include the identification, selection, and breeding of crops for salt tolerance. Furthermore, modern molecular biology approaches, including genetic engineering and mutation breeding, can be a valuable tool to tailor crop salt tolerance [3]. Nonetheless, time and monetary investment in the above-mentioned approaches can be a topic of discussion. Hence, agronomic approaches can serve as a time-efficient, inexpensive, and sustainable solution to mitigate the effects of salt stress in crop plants.

Various agronomic approaches and breeding programs have focused on imparting salinity tolerance in crops [4,6]. Among agronomic approaches, the use of allelopathic water extracts is gaining popularity because it is easily accessible, environmentally safe, and sustainable. Furthermore, these have been used to enhance the productivity of various crops [7,8]. Sorghum is a potential allelopathic crop exploited for the application of its extract owing to allelochemical presence. Nonetheless, it contains phenolics such as p-hydroxybenzoic acid, ferulic acid, and p-cumeric acid that, when used at low concentrations, enhance the growth of other crops [9]. The exogenous application of natural plant growth regulators such as aqueous extracts of sorghum and moringa enhances growth and development, photosynthesis, and the plant defense system, combating various abiotic stresses [10].

Organic fertilizers have been used in soil reclamation and for the enhancement of soil nutrient status for quite a long time [11,12]. It improves soil structure, increases mineral availability, enhances soil productivity and the quality of the produce, and has been reported as an economical approach to lower reliance on synthetic fertilizers [13]. Organic fertilizers and their derivatives enable plants to tolerate salt stress efficiently [14]. The cumulative activity of soil microbes and earthworms on any organic matter produces vermicompost. It is an organic fertilizer that possesses broader microbial activity and plant growth hormones and hence can be used to ameliorate the effect of salt stress in the soil–plant system [12]. Ever-increasing environmental degradation necessitates exploring sustainable and environmentally benign approaches to mitigate the detrimental effects of salinity on maize seedlings. The above scenario suggests much room for utilizing allelopathic water extracts and vermicompost to enhance crop yields in salinity-prone soils. Exploring the combined effect of the exogenous application of sorghum water extract (SWE) and soil-applied vermicompost for enhancing the growth and biomass production of maize grown under saline soils has rarely been reported in the literature. Keeping this in mind, the present research was conducted to evaluate the potential of vermicompost and sorghum water extract in mitigating salinity stress in maize seedlings.

## 2. Results

### 2.1. Morphological Parameters

The interactive effect of the factors (salinity, vermicompost, and SWE) under study was significant (*p* ≤ 0.05) in all growth attributes. Increasing salinity levels had a diminishing influence on the morphological attributes of maize. However, the application of vermicompost and sorghum water extract helped the maize seedlings to survive and grow under salinity, thereby depicting a positive effect on the morphological traits of maize.

#### Plant Biomass

**Figure 1:**
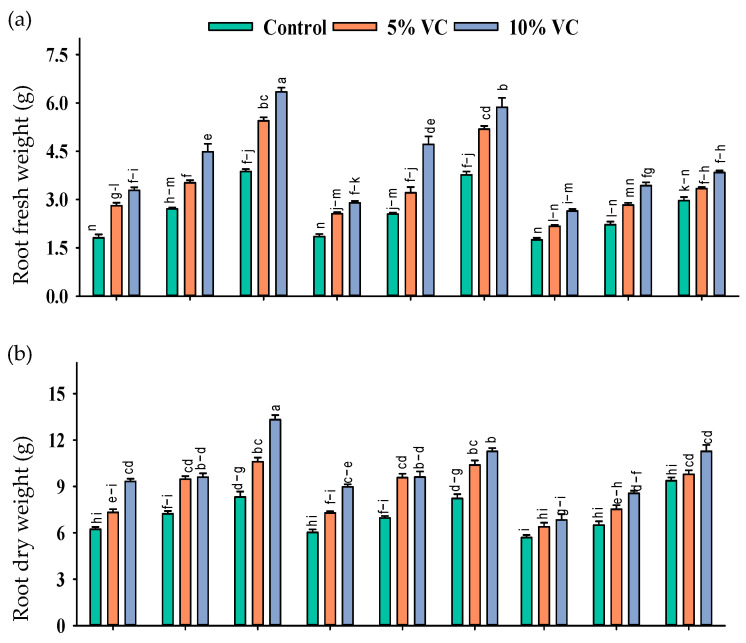

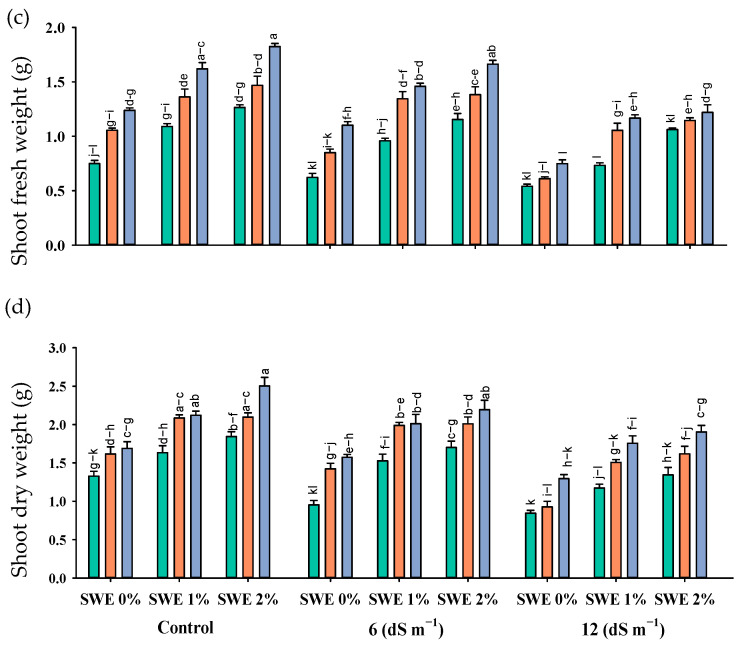
Root fresh weight (g; **a**), shoot fresh weight (g; **b**), root dry weight (g; **c**), and shoot dry weight (g; **d**) of maize as affected by various treatments of vermicompost (VC), sorghum water extract (SWE) under control, moderate salinity (6 dS m^−1^) and high salinity (12 dS m^−1^). Error bars depict the standard error of 3 replications (*n* = 3). Bars with the same letters do not differ significantly (*p* ≤ 0.05).

The application of vermicompost and SWE has a variable effect on maize seedlings grown under salinity. A positive effect of these treatments was recorded on the fresh and dry biomass of roots and shoots grown under salinity, implying that the exogenous application of SWE and soil-applied vermicompost effectively mitigated salt stress in maize seedlings (Table 1; Figure 1).

The mitigating effects of vermicompost and SWE were diluted at increasing salinity levels. As compared with the control, the application of 1% and 2% SWE improved root fresh weight by 77–186% and 160–229%, the root dry weight by 79–85% and 95–22%, shoot fresh weight by 54–67% and 54–81%, shoot dry weight by 136–152% and 149–155%, respectively, while with the application of 5 and 10% vermicompost, at moderate salinity of 6 dS m^−1^, respectively, the increases in these attributes were 18–84% and 89–84%, 41–53% and 56–63%, 21–50% and 37–51%, and 73–100% and 87–100%, at high salinity of 12 dS m^−1^.

### 2.2. Photosynthetic Pigment

**Figure 2:**
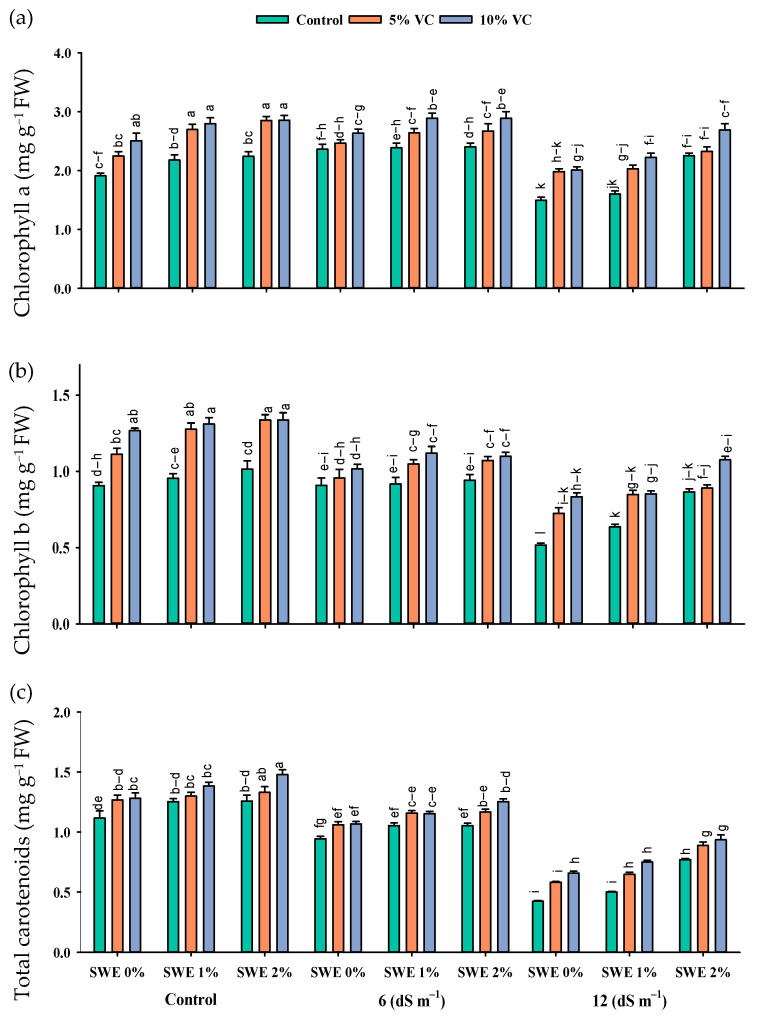
Chlorophyll a (mg g^−1^ FW; (**a**)), chlorophyll b (mg g^−1^ FW; (**b**)), and total carotenoids (mg g^−1^ FW; (**c**)) contents of maize as affected by various treatments of vermicompost (VC), sorghum water extract (SWE) under control, moderate salinity (6 dS m^−1^), and high salinity (12 dS m^−1^). Error bars depict the standard error of 3 replications (*n* = 3). Bars with the same letters do not differ significantly (*p* ≤ 0.05).

Salt stress reduced the maize chlorophyll contents as compared to the control. Nonetheless, vermicompost and foliar application of SWE enhanced the chlorophyll contents under salinity (Figure 2; Table 1). The interactive effect of vermicompost, SWE, and salinity (VC × SWE × S) on chlorophyll a and chlorophyll b was non-significant (*p* ≤ 0.05), except for total carotenoid. Plant samples were grown in salinity without applying vermicompost, and SWE showed a significant reduction in these pigments, while plants grown in salinity and receiving SWE and vermicompost showed maximum chlorophyll and carotenoid contents. At a salinity level of 6 dS m^−1^, the application of 1% and 2% SWE improved the chlorophyll a content by 4–5% and 13–14%, chlorophyll b by 2–4 and 1–5%, and carotenoid content by 4–5% and 3–12% under the application of 5 and 10% vermicompost, respectively, as compared to 12 dS m^−1^. Experimental results revealed that the maximum increase in chlorophyll contents under salinity was recorded at 2% SWE and 10% vermicompost.

### 2.3. Antioxidant Enzyme Activities

**Figure 3:**
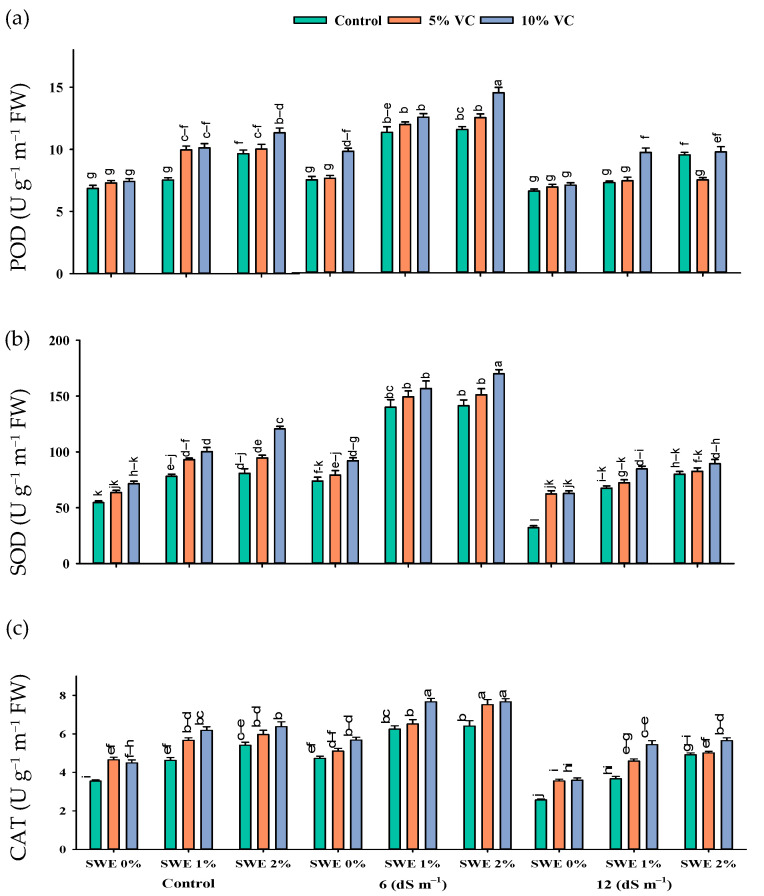
Activities of enzymatic antioxidants {POD (**a**), SOD (**b**), and CAT (**c**)} in maize are affected by various treatments of vermicompost (VC), sorghum water extract (SWE) under control, moderate salinity (6 dS m^−1^), and high salinity (12 dS m^−1^). Error bars depict the standard error of 3 replications (*n* = 3). Bars with the same letters do not differ significantly (*p* ≤ 0.05).

Antioxidant enzyme activity in maize was enhanced with the increasing levels of salinity. The interactive effect of vermicompost, SWE, and salinity (VC × SWE × S) on peroxidase (POD), superoxide dismutase (SOD), and catalase (CAT) activity varied significantly (*p* ≤ 0.05). The application of vermicompost and low-dose aqueous extract of sorghum in maize significantly improved the activities of antioxidant enzymes at moderate salinity (6 dS m^−1^) compared to the control and high salinity (12 dS m^−1^). POD activity was improved by the tune of 147.25%, SOD by 203.5%, and CAT by 107% at 6 dS m^−1^ salinity across vermicompost and foliar spray treatments. A reduction in the activities of these antioxidant enzymes was recorded under the control condition without the application of extract and organic amendments. However, soil-applied vermicompost and foliar-applied SWE improved the antioxidant enzyme activity in both the control and salinity levels of 6 and 12 dS m^−1^ (Figure 3; Table 1). Compared to the control (no application of vermicompost and SWE), the application of 2% SWE and 10% VC improved the activity of POD by 179 and 87%, SOD by 277 and 64%, and CAT by 116 and 59 at 6 and 12 dS m^−1^ salinity, respectively. Thus vermicompost and foliar spraying of SWE increased the activities of antioxidant enzymes in the control and high salt stress environments (Figure 3).

### 2.4. Leaf Malondialdehyde (MDA) and H_2_O_2_ Content

**Figure 4:**
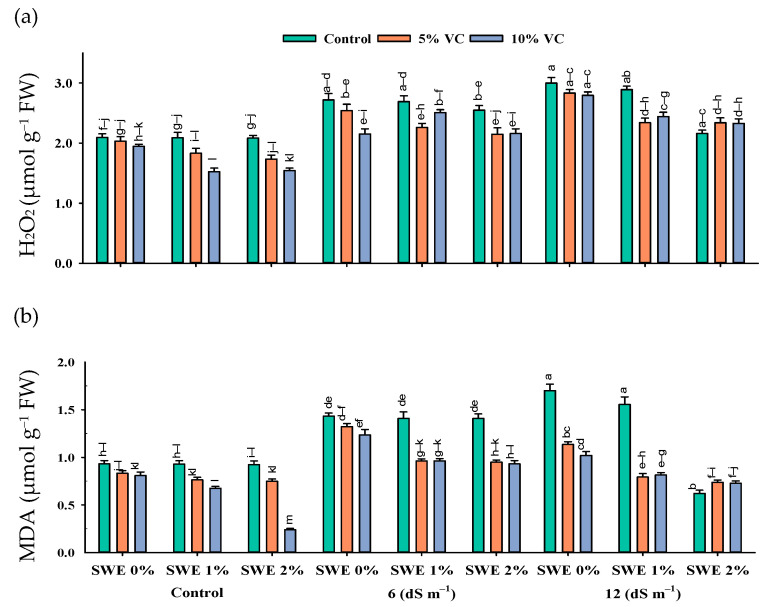
Hydrogen peroxide (H_2_O_2_) (µmol g^−1^ FW; (**a**) and malondialdehyde (MDA) (µmol g^−1^ FW; (**b**) contents in the leaf of maize as affected by various treatments of vermicompost (VC), sorghum water extract (SWE) under control, moderate salinity (6 dS m^−1^), and high salinity (12 dS m^−1^). Error bars depict the standard error of 3 replications (*n* = 3). Bars with the same letters do not differ significantly (*p* ≤ 0.05).

The interactive effect of salinity, vermicompost, and SWE on MDA and H_2_O_2_ contents was significant (*p* ≤ 0.05; Figure 4; Table 1). Plant samples grown in salinity without the application of SWE and vermicompost amendment showed the highest content of MDA and H_2_O_2_. However, applying vermicompost and aqueous extract of sorghum significantly reduced the harmful effects of salinity (Figure 4). At the high salinity of 12 dS m^−1^, vermicompost and foliar spraying of SWE decreased the MDA and H_2_O_2_ contents by 28–8% and 22–3%, respectively, compared with the respective control. Furthermore, the decrease was 3–1.85% and 3–0.05%, respectively, at a moderate salinity of 6 dS m^−1^.

### 2.5. Mineral Ion Contents

**Figure 5:**
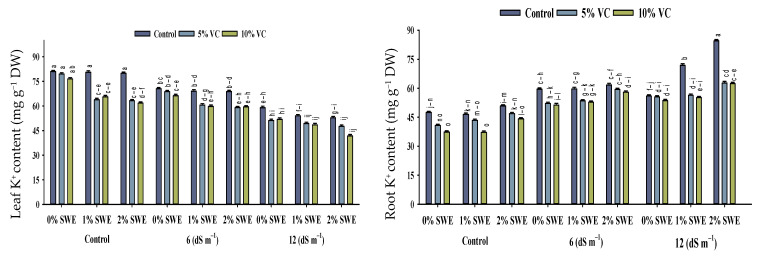

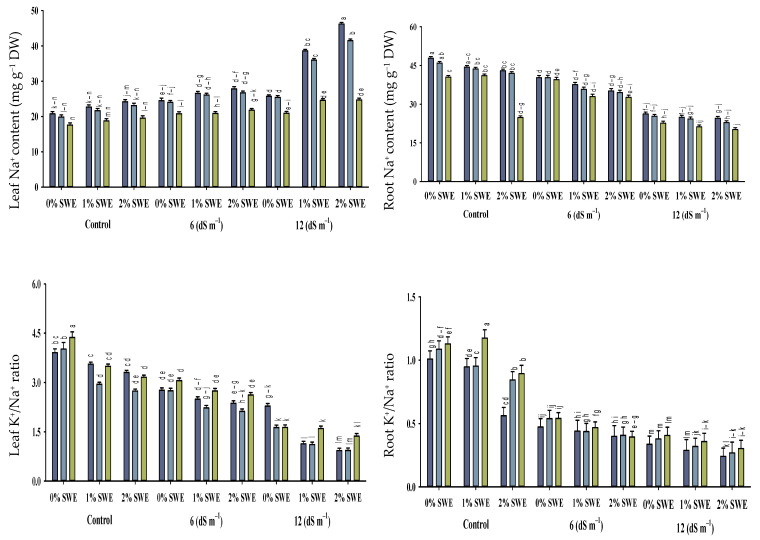
Sodium (Na^+^) and potassium ion (K^+^) contents and K^+^/Na^+^ ratio of maize as affected by various treatments of vermicompost (VC), sorghum water extract (SWE) under control, moderate salinity (6 dS m^−1^), and high salinity (12 dS m^−1^). Bars with the same letters do not differ significantly (*p* ≤ 0.05).

Salinity stress, vermicompost, and SWE treatment significantly impacted the concentrations of Na^+^, K^+^, and the K^+^/Na^+^ ratio in various parts of maize (Figure 5; Table 1). The interactive effects of vermicompost, SWE, and salinity (VC × SWE × S) on Na^+^ and K^+^ concentrations and the K^+^/Na^+^ ratio in the shoots and roots of maize were found to be statistically significant (*p* ≤ 0.05). Salinity disturbed the ionic balance and significantly increased the accumulation of Na+ in plant parts compared to unstressed plants. While it decreased the K^+^ concentration and K^+^/Na^+^ ratio in maize shoots and roots, the highest reduction in K^+^ concentration was observed in severe salinity.

#### 2.5.1. Na^+^ and K^+^ Contents

Salt stress of 12 dS m^−1^ enhanced the concentration of Na^+^ ion in maize shoots to the tune of 75–196% and in roots by 19–35%. However, vermicompost and foliar spray significantly (*p* ≤ 0.05) decreased the concentration of sodium Na^+^ under salinity. Variations were recorded between vermicompost and SWE for sodium ion Na^+^ concentration. Vermicompost and foliar spraying of SWE-treated plants showed less concentration of Na^+^ ions in shoots. The concentration of K^+^ ions in maize shoots and roots differed statistically (*p* ≤ 0.05) in response to salinity and vermicompost and foliar spray treatments. Overall, salinity decreased the concentration of potassium ion K^+^ in maize shoots and roots. However, soil-applied vermicompost and foliar spraying of SWE significantly enhanced the concentration of K^+^ ions in maize shoots and roots under the control and salinity conditions. Compared with the control, vermicompost and SWE-applied plants showed maximum potassium K^+^ contents in shoots of 56–60, and 25–30% compared with roots.

#### 2.5.2. Maize K^+^/Na^+^ Ratio

The increased concentration of salinity decreased the ratio of K^+^/Na^+^ in maize seedlings. The application of vermicompost and foliar spraying of SWE enhanced the K^+^/Na^+^ ratio of maize shoots and roots under control and salinity conditions. Soil-applied vermicompost at 5 and 10% and foliar-applied SWE at 1 and 2% improved K^+^/Na^+^ ratio by 56–60 and 25–30%, and 52–41% and 54–43%, respectively, at 6 dS m^−1^, while at high salinity, these treatments were not very effective in increasing K^+^/Na^+^ ratio. Thus, soil-applied vermicompost and the exogenous application of SWE maintained ionic balance and reduced salinity-induced harmful effects.

### 2.6. Principal Component Analysis

**Figure 6:**
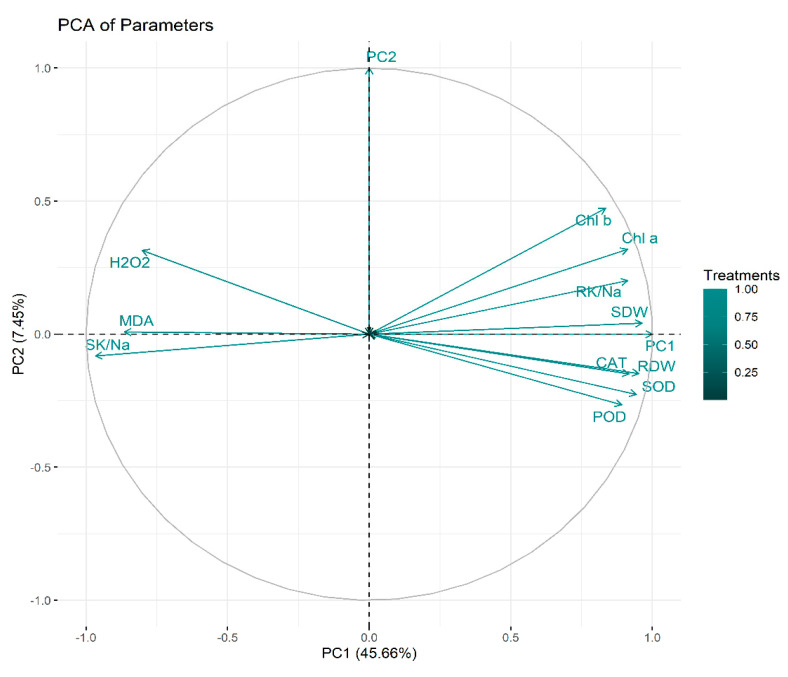
Principal component analysis. RDW: root dry weight (g), SDW: shoot dry weight (g), Chl a: chlorophyll a contents (mg g^−1^ FW), Chl b: chlorophyll b (mg g^−1^ FW), POD: peroxidase activity (U g^−1^ m^−1^ FW), SOD: superoxide dismutase activity (U g^−1^ m^−1^ FW), CAT: catalase activity (U g^−1^ m^−1^ FW), MDA: malonaldehyde contents (µmol g^−1^ FW), H_2_O_2_: hydrogen peroxide contents (µmol g^−1^ FW), SK/Na: shoot potassium/sodium ratio, RK/Na: root potassium/sodium ratio.

**Figure 7:**
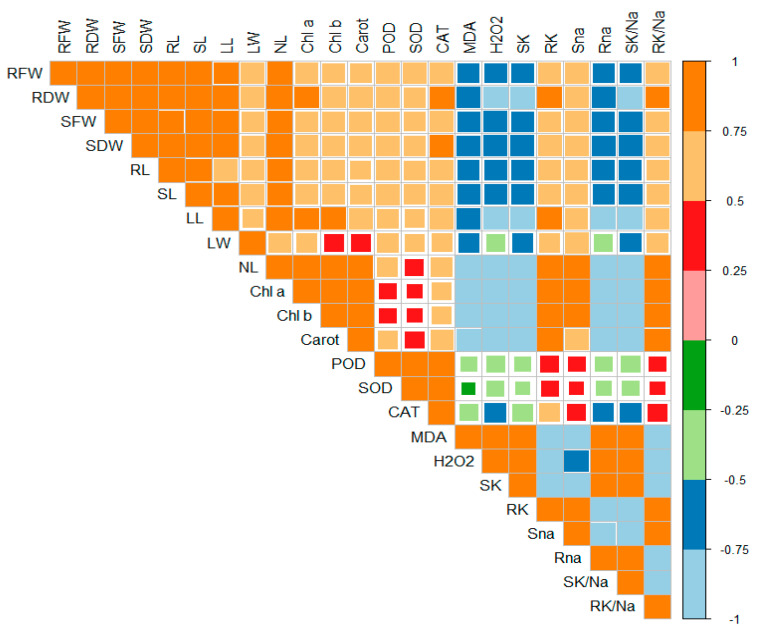
Correlation matrix represents the relationships between various parameters under study. RFW: root fresh weight (g), RDW: root dry weight (g), SFW: shoot fresh weight (g), SDW: shoot dry weight (g), Chl a: chlorophyll a contents (mg g^−1^ FW), Chl b: chlorophyll b (mg g^−1^ FW), Carot: total carotenoids (mg g^−1^ FW), POD: peroxidase activity (U g^−1^ m^−1^ FW), SOD: superoxide dismutase activity (U g^−1^ m^−1^ FW), CAT: catalase activity (U g^−1^ m^−1^ FW), MDA: malonaldehyde contents (µmol g^−1^ FW), H_2_O_2_: hydrogen peroxide contents (µmol g^−1^ FW), SK: shoot potassium content (mg g^−1^ DW), RK: root potassium content (mg g^−1^ DW), Sna: shoot sodium content (mg g^−1^ DW), Rna: root sodium content (mg g^−1^ DW), SK/Na: shoot potassium/sodium ratio, RK/Na: root potassium/sodium ratio.

Principal component analysis (PCA) was executed to determine the role of vermicompost and SWE in salinity stress alleviation in maize seedlings. PC1 and PC2 were the two components that were subjected to PCA. The share of PC1 and PC2 in total variance was 45.66 and 7.45%, respectively (Figure 6). The positive and negative correlations between various variables under study are depicted in Figure 7. From this it is clear that H_2_O_2_ and MDA contents are negatively correlated with the plant biomass attributes, antioxidant enzyme activities, and chlorophyll contents.

## 3. Discussion

Salinity is the main growth-limiting factor and a menace to crop production. Saline soils contain high levels of soluble salts, causing damage to the plant by reducing the uptake of nutrients and disturbing their water relations. Plant growth and development are inhibited by salinity, causing a drastic decrease in production, which is linked with a decrease in photosynthesis efficiency [15].

In the present study, salinity stress decreased the morphological traits of maize seedlings (Figure 1). Salinity reduced the fresh and dry weight of maize plants. Shoot and root length are sensitive to salt stress [16]. The size and efficiency of a plant’s photosynthetic machinery are reduced under salinity, and adversities continue to bring cellular damage related to growth retardation in plants [17,18]. However, applying SWE and vermicompost helped plants cope with the detrimental effects of salinity, and plants depicted better growth and increased biomass. Sorghum and brassica water extracts helped wheat plants to maintain normal growth under abiotic stresses [9,19]. Furthermore, the application of vermicompost decreased the effects of soil salinity in *Foeniculum vulgare* and *Suaeda salsa* plants and improved soil health [12,20], suggesting that the secondary metabolites present in plant water extracts have a beneficial role in alleviating the effect of salinity.

Photosynthetic components, including chlorophylls and carotenoids, are also affected by salt stress. In the present experiment, chlorophyll a, b, and total carotenoid contents showed significant reductions under salinity (Figure 2). The application of vermicompost and spraying of SWE helped the maize seedlings cope with the detrimental effects of salinity, which resulted in an improvement in photosynthetic pigment concentration. A previous study documented the enhancement in the photosynthetic pigment of maize by the application of a low dose of SWE under salinity [21]. Studies have already documented the beneficial role of vermicompost application to plants grown under salinity, where the application of vermicompost helped the plants to grow and function normally under a salt stress environment [22].

Salt stress causes the excessive production of ROS such as hydrogen peroxide, superoxide, and singlet oxygen, which induces phytotoxic reactions such as lipid peroxidation [23]. H_2_O_2_ is the product of the metabolism of the cells. It is produced in large amounts in chloroplast, mitochondria, and peroxisomes. In plants, the increased accumulation of H_2_O_2_ caused oxidative damage and led to cell death; as a result, plant biomass decreased. Increasing salt stress also enhanced the concentration of MDA, which is the product of lipid peroxidation. The results of the present study showed that salinity enhanced the production of H_2_O_2_ and MDA (Figure 4). A negative correlation was observed between H_2_O_2_ and MDA contents and overall plant growth parameters (Figure 7). However, the combined beneficial effects of vermicompost and SWE reduced the production of H_2_O_2_ and cell injury, as documented in the present study. Kamran et al. [21] worked on the effect of SWE on the growth, physiology, and photosynthetic activity of maize seedlings and observed that foliar application of a low dose of SWE decreased the concentration of MDA in maize.

The present study recorded a reduction in chlorophyll and carotenoid contents under salinity stress, which ultimately disturbed plant photosynthetic efficiency. A high rate of respiration, ionic toxicity, the permeability of the membrane [24], and lower photosynthetic efficiency are some of the major consequences of salt stress in plants [25]. Kwon et al. [26] reported that under salt stress, lower stomatal conductance is responsible for the decrease in the efficiency of photosynthesis. A lower uptake of salts by the application of vermicompost in soil and the exogenous application of osmoprotectants saved the plant from damage caused by salinity, primarily by decreasing the production of ROS.

The increased production of ROS triggers the antioxidant enzymes in plants to provide a defense shield against oxidative damage. However, the higher and prolonged stress levels decrease the efficiency of antioxidants because ROS production outstrips the generation of antioxidant enzymes [27,28], and the same happens under salinity. Overproduction of ROS is toxic to carbohydrates, lipids, and protein. The present results showed a significant improvement in the activities of antioxidant enzymes (SOD, POD, and CAT) under salinity (Figure 4), and this increase was in the order of 6 dS m^−1^ > control > 12 dS m^−1^. The antioxidant enzyme activities helped the plants to scavenge the ROS. They maintained normal growth as antioxidant enzyme activities and plant biomass production was correlated positively (Figure 7). Improved antioxidant enzyme activities were recorded in maize under a saline environment to cope with the oxidative damage from ROS [29]. In the present study, the application of 2% SWE along with 10% vermicompost improved antioxidant enzyme activities and diminished the negative impacts of salinity on maize. Our results are also similar to past studies where the low-dose application of SWE increased the antioxidant enzyme activities [21].

In the present study, an elevated Na^+^ ion concentration was observed in plant parts under salinity stress, which led to ionic imbalance and decreased the K^+^ uptake in maize seedlings. In saline soils, high levels of sodium and chloride ions in the soil system caused a severe nutrient imbalance in maize due to their interference with the uptake of other nutrients such as phosphorous, potassium, calcium, iron, magnesium, and nitrogen [30]. In plants, a high level of Na^+^ causes nutritional imbalance and ionic toxicity [31]. The deposition of intracellular Na^+^ ions during salt stress alters the K–Na ratio, which appears to impact photosynthetic bioenergetic systems. Salinity caused an increase in the concentration of Na^+^ and a reduction in the concentration of K^+^ and the K^+^/Na^+^ ratio (Figure 5). It is observed that inclined levels of NaCl reduced the potassium level due to the membrane’s depolarization by Na^+^ ions [32]. Therefore, reducing sodium concentration in cytosol and improving the level of the K^+^/Na^+^ ratio in cells is critical for salt tolerance. The present study’s findings are similar to Tuna et al. [33], where salinity decreased potassium ions’ uptake, and its level in the salt-stressed plants was lowered more than in unstressed plants. Under salinity, competition between the uptake of Na^+^ and K^+^ occurred in the plants leading to a low concentration of K^+^ inside the plant [34]. A high concentration of Na^+^ is very toxic and reduces the growth of plants (Figure 1). Eker et al. [35] worked on the consequences of salinity stress on the production of dry matter and ion accumulation in maize. They reported a decrease in potassium content both in roots and leaves, but the concentration of K^+^ in roots was more decreased than in leaves. However, the application of vermicompost and SWE resulted in the reduced uptake of Na^+^ and increased K^+^ level in the root and shoot (Figure 5). Resultantly, an improved K^+^/Na^+^ ratio was observed in maize seedlings after the application of vermicompost and SWE. Under salt-affected soils, the application of vermicompost was associated with improved nutrient uptake and ionic balance in plants [22,36].

**Figure 8:**
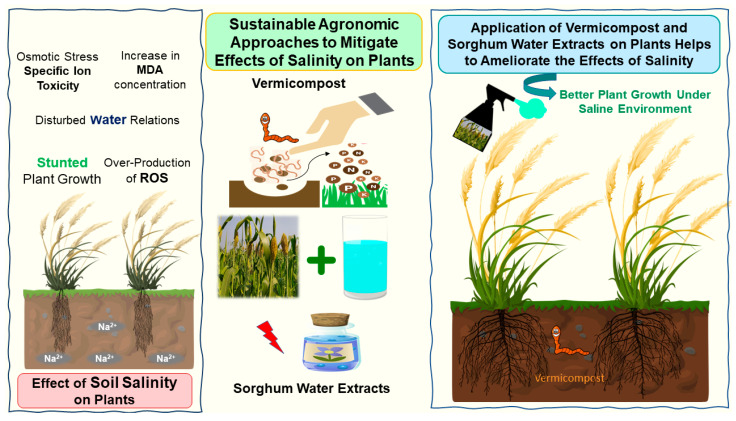
Graphical illustration of the effect of salinity on plants and sustainable agronomic options to mitigate it.

Numerous studies have documented the role of vermicompost and plant water extract in mitigating the adversities of salinity in plants. However, the present study demonstrated the synergistic role of both the strategies (vermicompost and plant water extracts) to cope with the detrimental effects of salinity on maize seedlings. Figure 8 depicts the graphical illustration of the present study.

## 4. Materials and Methods

### 4.1. Location of the Experiment

The pot study was conducted in February 2021 at Warehouse, Department of Agronomy, University of Agriculture, Faisalabad, Pakistan (31° N, 73° E). The soil was sandy loam and was examined for physicochemical properties, i.e., pH, EC, and organic matter (Table 2). Seeds of maize hybrid (DK 9108 Pioneer, Des Moines, IA, USA) were procured from the local representative of Pioneer Pakistan (Pvt.) Ltd., and the germination percentage of the seed was determined before sowing.

### 4.2. Sorghum Water Extract Preparation

The preparation of SWE was carried out following Khaliq et al. [37]. Mature sorghum plants (*Sorghum bicolor* L.) were harvested from the Agronomy Farm, University of Agriculture Faisalabad. Collected plants were shade dried for a few days and chopped into pieces. The plant material was soaked in distilled water for 24 h at ambient temperature in a 1:10 (*w*/*v*) ratio. After 24 h the extract was filtered using a sieve. It was designated as 100% SWE (stock solution) and dilutions of 1 and 2% of SWE prepared thereof. The osmotic potential of the prepared solutions was recorded with an osmometer (VAPRO-5520, WESCOR, Inc., Logan, UT, USA). The SWE at 1 and 2% recorded an osmotic potential of −0.680 MPa (272 mmol kg^−1^) and −0.695 MPa (278 mmol kg^−1^), respectively. Further characterization of sorghum allelopathic extract has already been reported in the literature [37,38].

### 4.3. Experimental Setup

The experiment consisted of 3 factors; each factor has different levels, i.e., salinity (control, 6, and 12 dS m^−1^), vermicompost (control, 5, and 10%), and aqueous extract of sorghum (control, 1, and 2%), and was laid on completely randomized design having three replications in the warehouse. Ten plants were maintained in each pot. Foliar application of extract was performed with the help of a laboratory pressure sprayer (DADA sprayers, Lahore, Pakistan) after 25 days of sowing.

### 4.4. Imposition of Salinity

Soil electrical conductivity (EC) was calculated by a conductivity meter (HI-9811, Hanna Instruments, Woonsocket, SD, USA) and was used to calculate total soluble salts (TSS). Soil saturation paste was prepared by presenting a measured quantity of water into a measured amount of soil. The saturation percentage was calculated as follows

$$\mathrm{Saturation}\%=\frac{B-C}{C-A}\times 100$$

where A is the weight of the empty china dish (g), B is A plus weight of the china dish inclusive of saturated soil paste (g), and C is B after oven drying (g). The measured EC was subtracted from the desired salinity level and multiplied by 10 to impose that level. The formula for salinity imposition in pots is given as

$$\mathrm{Salt}\;\mathrm{required}\;\left({g\;\mathrm{kg}}^{-1}\right)=\frac{\mathrm{TSS}\;\times \;\mathrm{molecular}\;\mathrm{weight}\;\mathrm{of}\;\mathrm{salt}\;\times \;\mathrm{soil}\;\mathrm{saturation}\%}{100\times 1000}$$

### 4.5. Crop Husbandry

Ten maize seeds were sown at equidistance per pot. The pot size was 18 × 17 cm, and each pot was filled with 5 kg of soil. Vermicompost was collected from the Vermicompost Center, University of Agriculture, Faisalabad, Pakistan [39]. The dose of vermicompost as per treatments was mixed in the soil at the pot filling before sowing. NPK at recommended rate was added to the soil as a basal dose. All other practices, except treatments, were kept the same.

### 4.6. Measurement of Morphological Parameters

Morphological parameters were recorded at the seedling harvest stage (45 days after sowing). Three plants were collected per pot and their fresh shoot and root biomass were determined by using an electric weighing balance. A constant dry weight of plant samples was achieved by air-drying followed by oven drying at 70 °C and measured afterwards.

### 4.7. Determination of Photosynthetic Pigments

For determination of photosynthetic pigment (chlorophyll a, chlorophyll b, and total carotenoid) leaf samples at final harvest were processed as per respective protocols. The leaf sample (0.5 g) was ground in 80% acetone. The solution was centrifuged for 20 min at 13,000× *g* rpm after filtration. Filtrate absorbance was determined at 663, 645, and 480 nm [23].

### 4.8. Determination of Enzymatic Antioxidant Activities

For the determination of antioxidant enzymes, leaf samples at harvest were taken and stored at −80 °C in the zipper bag. Antioxidant enzyme assay was conducted following Khaliq et al. [23]. A reaction mixture of guaiacol (10 mM), H_2_O_2_ (5 nM), and phosphate buffer (50 nM; pH 7.0) was prepared and preheated at 20 °C to determine POD activity. Then 0.2 mL enzyme + 2.8 mL reaction solution was mixed and added to a centrifuge tube. The variation in absorbance of the reaction mixture was recorded at 470 nm at 30 s for 5 min.

For CAT activity, a 100 mM H_2_O_2_ and phosphate buffer (50 mM; pH 7.8) reaction mixture was prepared and kept at 25 °C in a water bath. Phosphate buffer (0.2 mL) and enzyme solution (0.2 mL) were added to the test tube and heated for 3 min in the water bath. Then 0.3 mL solution (100 mM H_2_O_2_) was added to test tubes. After mixing, the change in absorbance was recorded and observed every 20 s for 5 min at 240 mm by a UV–visible spectrophotometer (UV-4000, ORI, Hille, Germany).

The activity of SOD was estimated by preparing an extraction buffer {EDTA (5 mM) + phosphate buffer (0.1 M)}. The leaf sample (100 mg) was ground using a pestle and mortal in 5 mL extraction buffer and then centrifuged at 4 °C for 10 min at 10,000× *g* rpm. The reaction mixture (3 mL) was prepared by adding EDTA (0.1 mL; 3 mM) + sodium carbonate (0.1 mL) + potassium phosphate buffer (1.5 mL; 100 mM) + methionine (0.2 mL; 200 mM) + NBT (0.1 mL; 2.25 mM) + distilled water (1 mL) + riboflavin (0.1 mL), and enzyme (0.1 mL) was taken in test tubes. The reaction was initiated by placing the reaction mixture under the 30 W illumination of fluorescent lamps. The reaction was begun by turning on the fluorescent lamps and stopped after 5 min. The NBT photo-reduction produced blue formazan in tubes that contained enzyme extract. A blank solution containing the same reaction mixture (excluding enzyme extract) did not produce color. Absorbance was observed at a wavelength of 560 nm by a UV–visible spectrophotometer.

### 4.9. Determination of Reactive Oxygen Species (ROS)

#### H_2_O_2_ and MDA Contents

H_2_O_2_ and MDA contents were determined by following Velikova et al. [40] and Bailly et al. [41], respectively. To determine H_2_O_2_ contents, fresh leaves weighing 500 mg were homogenized with TCA (5 mL; 0.1% *w*/*v*) in a pestle and mortar (prechilled) and centrifuged for 15 min at 12,000× *g* rpm. The supernatant (0.5 mL) was mixed in potassium iodide (KI; 1 mL; 1 M) and phosphate buffer (0.5 mL; 0.05 M; pH 7.0). The mixture was shaken in a vortex, and absorbance was recorded at 390 nm in UV–visible spectrophotometer by taking water as a blank. For MDA determination, extraction was performed by homogenizing 0.5 g leaf with TCA (5 mL; 1% *w*/*v*), and the homogenate was centrifuged at 15,000× *g* rpm for 15 min. Then, 3 mL of 0.5% thiobarbituric acid prepared in 20% TCA (*w*/*v*) was added to 1 mL of the supernatant. The mixture was boiled for 15 min, cooled on ice, and centrifuged at 15,000× *g* rpm for 5 min. The supernatant was used for MDA determination. Absorption of the extracts at 450, 532, and 600 nm was determined at a UV–visible spectrophotometer (UV-4000, ORI, Germany).

### 4.10. Determination of Mineral Ion (Na^+^ and K^+^) Concentration

Leaf Na^+^ and K^+^ were determined by a flame photometer (Model 360, Sherwood, UK) [42]. Young, fully expanded leaves and root samples were collected and weighed for fresh and oven-dried weight. Dried samples (weighing less than 0.25 g) were digested in a 1% nitric acid (HNO_3_) solution. The 1% HNO_3_ solution (25 mL) was collected in falcon tubes and digested on a hot plate at 85 °C for 4 h. After digestion, 1 mL of the digestion mixture (from 25 mL solution) was diluted with deionized water to 10 mL. The prepared sample was run on a flame photometer to determine Na^+^ and K^+^.

### 4.11. Statistical Analysis

The normal distributions of data were checked and were found to be in permissible ranges. Three-way ANOVA was constructed and used SPSS 24 package (SPSS, Chicago, IL, USA). The mean sum of square component of ANOVA for each factor and possible interaction/s are presented in Table 1. Tukey’s HSD test was employed at a 5% probability level to find the significance of treatment means. Graphs were prepared in Sigma plot 14.0 (SPSS Inc., Chicago, IL, USA). The correlation matrix and principal component analysis were carried out in R Studio 4.6.1 (R Studio, Boston, MA, USA).

## 5. Conclusions

Salinity-induced production of reactive oxygen species and uptake of Na^+^ ions caused cellular damage and ionic imbalance, which restricted growth having negative implications for the morphological and biochemical attributes of maize. Nonetheless, the combined beneficial application of vermicompost and sorghum water extract mitigated such harmful effects of salinity. The beneficial effect of SWE was more pronounced at 2% and of vermicompost at 10% levels. Application of vermicompost and SWE improved the seedling growth of maize owing to increased photosynthetic pigments and activities of antioxidants (SOD, POD, and CAT). It also upsurged the uptake of potassium ions in maize seedlings. This study demonstrated biochemical and ionic bases for the improved seedling growth of maize due to the combined effect of soil-applied vermicompost and foliar-applied SWE grown under salt stress.

## Figures and Tables

**Table 1:** Mean sum of square summary related to the effect of vermicompost and foliar-applied sorghum water extract on morphological attributes, photosynthetic pigments, enzymatic antioxidants, ionic contents, and H_2_O_2_ and malondialdehyde (MDA) contents.

	Mean Sum of Square					
Characteristics	Salinity	VC	SWE	Salinity × VC	Salinity × SWE	Salinity × VC × SWE
Root fresh weight (g)	11.02 **	21.77 **	24.10 **	2.11 **	1.00 **	0.33 **
Root dry weight (g)	1.25 **	1.66 **	1.35 **	0.02 **	0.05 **	0.03 **
Shoot fresh weight (g)	22.97 **	48.01 **	59.82 **	1.71 **	2.00 **	1.47 **
Shoot dry weight (g)	1.53 **	2.93 **	4.04 **	0.05 **	0.07 **	0.05 **
Chlorophyll a (mg g^−1^ FW)	6.70 **	0.55 **	1.35 **	0.05 **	0.08 **	0.01 ^ns^
Chlorophyll b (mg g^−1^ FW)	1.84 **	0.11 **	0.46 **	0.006 ^ns^	0.04 **	0.01 ^ns^
Total carotenoid (mg g^−1^ FW)	4.22 **	0.29 **	0.68 **	0.04 **	0.20 **	0.044 **
Peroxidase (U g^−1^m^−1^FW)	123.37 **	103.97 **	34.59 **	3.31 **	0.66 *	0.95 **
Superoxide dismutase (U g^−1^m^−1^FW)	29,908.5 **	17,737 **	5092.1 **	2478.6 **	150.7 **	240.7 **
Catalase (U g^−1^m^−1^FW)	33.93 **	22.47 **	11.91 **	0.25 *	0.21 *	0.18 *
Leaf Na^+^ (mg g^−1^ DW)	4592.72 **	511.36 **	770.07 **	225.86 **	189.59 **	34.47 **
Leaf K^+^ (mg g^−1^ DW)	3289.3 **	447.81 **	694.25 **	24.37 **	26.68 **	23.68 **
Leaf K^+^/Na^+^ ratio	36.33 **	4.94 **	7.38 **	0.57 **	0.58 **	0.04 *
Root Na^+^ (mg g^−1^ DW)	2905.5 **	442.88 **	858.07 **	35.65 **	113.85 **	23.84 **
Root K^+^ (mg g^−1^ DW)	3400.81 **	121.68 **	218.18 **	33.74 **	39.3 **	22.78 **
Root K^+^/Na^+^ ratio	2.66 **	0.22 **	0.42 **	0.025 **	0.02 **	0.008 **
H_2_O_2_ (µmol g^−1^ FW)	5.03 **	0.79 **	2.48 **	0.10 **	0.45 **	0.04 **
MDA (µmol g^−1^ FW)	4.22 **	0.48 **	1.20 **	0.06 **	0.03 ^ns^	0.04 **

*p* ≤ 0.01 = ** *p* ≤ 0.05 = *, *p* ≥ 0.05 = ^ns^; VC = vermicompost; SWE = sorghum water extract.

**Table 2:** Soil analysis report.

Chemical Analysis	
ECe	1.64 dS m^−1^
pHs	7.83
SAR	12.02
TSS	45.05 m·mol L^−1^
Na^+^	30.95 m·mol L^−1^
K^+^	0.45 m·mol L^−1^
Cl^−^	20.88 m·mol L^−1^
Soil organic carbon	2.54 g kg^−1^ soil
Physical analysis	
Sand	46%
Silt	30%
Clay	25%
Textual class	Sandy loam

## Data Availability

Not applicable.
